# Among friends: a qualitative exploration of the role of peers in young people's alcohol use using Bourdieu's concepts of habitus, field and capital

**DOI:** 10.1111/1467-9566.12467

**Published:** 2016-08-30

**Authors:** Georgie J. MacArthur, Nina Jacob, Pandora Pound, Matthew Hickman, Rona Campbell

**Affiliations:** ^1^ School of Social and Community MedicineUniversity of BristolUK; ^2^ Cardiff University School of Social SciencesCardiffUK

**Keywords:** adolescence, young people, young adult, alcohol, binge drinking, peer, Bourdieu, qualitative

## Abstract

Drinking is viewed by young people as a predominantly social activity which provides an opportunity for entertainment and bonding with friends. Using Bourdieu's concepts of habitus, field and capital, this article explores young people's attitudes and beliefs around alcohol use, influences on behaviour, and the role of peers, with a view to informing the development of preventive interventions. Semi‐structured interviews were conducted with 28 young people aged 18–20 in the south west of England. We describe how friends were integral in drinking experiences, and drinking with friends was equated with fun and enjoyment. In this way, the desire for social and symbolic capital appeared to be a key motivator for adolescent drinking. Critically, however, wider cultural norms played the predominant role in shaping behaviour, via the internalisation of widely accepted practice and the subsequent externalisation of norms through the habitus. Applying Bourdieu's theory suggests that population‐level interventions that regulate alcohol consumption, and thus disrupt the field, are likely to facilitate behaviour change among young people by driving a response in habitus.

## Introduction

Alcohol consumption rates among young adults in the UK are some of the highest in Europe (Hibell *et al*. 2011). Although the proportion of young people that report drinking alcohol has fallen slightly over recent years, a substantial proportion continues to engage in heavy episodic drinking. Over 70 per cent of 15–16 year olds report drinking monthly and one third report hazardous drinking (Bremner *et al*. 2011, MacArthur *et al*. 2012). Liberalised licensing laws and expansion of the night‐time economy have helped to embed alcohol consumption into social culture for young adults. Alongside such changes, alcohol‐related hospital admissions have risen steadily over the past decade (Health and Social Care Information Centre 2015, Public Health England 2016) and over 70 per cent of accident and emergency attendances may be alcohol related at weekends (Parkinson *et al*. 2015). These trends, alongside evidence that adolescent drinking is associated with injury, violence, antisocial behaviour, risky sexual behaviour, adverse neurological consequences and adult alcohol dependence (Bava and Tapert 2010, Rehm *et al*. 2012, Shield *et al*. 2012, Viner and Taylor 2007), highlight the public health importance of understanding and preventing harmful alcohol use behaviour in young people.

Despite the potential consequences of alcohol consumption, however, drinking remains integral to social events and social culture for many young adults, with the primary goal being entertainment, excitement, having fun, and bonding with friends (de Visser *et al*. 2013, Niland *et al*. 2013, Percy 2011, Szmigin *et al*. 2008). Studies to date report a ‘culture of intoxication’ for many young people, involving the active pursuit of drunkenness (Percy *et al*. 2011, Roberts *et al*. 2012, Sondhi and Turner 2011), albeit through a ‘calculated hedonism’ or ‘controlled loss of control’ (Measham and Brain 2005, Szmigin *et al*. 2008), reflected in drinking customs that evolve within friendship groups to facilitate enjoyment and shared consumption (Järvinen and Gundelach 2007, Percy *et al*. 2011).

While adolescent drinking culture may be shaped by the extent of monitoring and supervision by parents, parental role modelling, and perceptions around social norms (Jacob *et al*. 2015, Kelly *et al*. 2012, Sondhi and Turner 2011), peers also play a vital role, via their actual and perceived drinking behaviour and via the predominantly social context of alcohol consumption (de Visser *et al*. 2013, Niland *et al*. 2013, Szmigin *et al*. 2008). Quantitative studies report a greater likelihood of individual drinking associated with an increase in the number of drinking peers, which may be mediated by both peer influence and/or peer selection (Ali and Dwyer 2010, Bot *et al*. 2005, Fujimoto and Valente 2012, Kelly *et al*. 2012, Mercken *et al*. 2012). Furthermore qualitative studies highlight the integral nature of friends to young people's drinking experiences and enjoyment of nights out, the importance of the social setting, and the friendship group culture (‘idioculture’) (Lunnay *et al*. 2011, Percy *et al*. 2011, Roberts *et al*. 2012, Sheehan and Ridge 2001).

Despite such findings however, there remains a need for an understanding of the views of young people in relation to alcohol consumption; the social context of drinking; the development and impact of different drinking cultures; and the effects of peer norms and peer alcohol use, to inform the development of preventive interventions. While the prevention of harm associated with alcohol use in young people is a critical issue in public health (Newbury‐Birch *et al*. 2008), there remain a number of gaps in the evidence base relating to effective interventions during adolescence (Foxcroft and Tsertsvadze 2012, Spoth *et al*. 2008).

Bourdieu's theory has been applied to alcohol research by others (Brierley‐Jones *et al*. 2014, Järvinen and Gundelach 2007, Lunnay *et al*. 2011, Townshend 2013), who have highlighted the role of social, cultural and symbolic capital, and distinction, in influencing behaviour among adolescent and adult drinkers. However, Bourdieu's theory has not yet been applied to qualitative data relating specifically to peer influences on alcohol drinking with a view to aiding understanding of potentially effective preventive interventions. In this paper, we apply Bourdieu's concepts of habitus, field and capital (Bourdieu and Wacquant 1992) to help to explain the comparative roles of friends and the wider drinking culture in shaping adolescent alcohol use behaviour, and to highlight potential public health approaches that could be implemented to prevent alcohol‐associated harm.

## Theoretical approach

The concepts of habitus, field and capital developed by the French sociologist Pierre Bourdieu are particularly helpful in the exploration of alcohol use behaviour, owing to Bourdieu's focus on, and explanation of, the social world and the dispositions that shape behaviour, thoughts and feelings in social contexts. Critically, Bourdieu used these concepts to emphasise the interaction between agency and structure, bringing micro‐level and macro‐level factors together to explain behaviour. This is particularly relevant in examining alcohol use, which is shaped by multiple factors, including individual psychological propensities and wider forces in the social environment, and which is primarily acted out in the social context (Kokotailo 2010).

### Habitus, field and capital

Bourdieu describes habitus as a ‘structuring and structured structure’, which is structured by past and present circumstances (such as family context); structuring in that it helps to shape behaviour; and a structure in itself, because it is comprised of a system of dispositions which generate tastes and inclinations (Grenfell 2008). Habitus is ‘a way of being, a habitual state (especially of the body) and, in particular, a disposition, tendency, propensity, or inclination’ (Bourdieu 1977: 214), and a structure that ‘at every moment structures new experiences in accordance with the structures produced by past experiences’ (Bourdieu 1990: 60). In this way, habitus helps to explain the predictability of social life, since it gives rise to new experiences based on the internalisation of previous occurrences and events.

The habitus also explains wider practice, through its interaction or ‘unconscious relationship’ with what Bourdieu describes as a ‘field’ (Grenfell 2008). A field is a boundaried social space with its own principles, in which actors struggle or compete to change or preserve its boundaries and form in line with their interests. Bourdieu has likened the field to a game, although the rules are not explicitly defined. Individuals in a field have an investment in the game (*illusio*); they believe in the game and its stakes (*doxa)*; and through their involvement they agree that the game is worth playing (*collusion)*. In other words, all of the actors have awareness of, and adhere to, the ‘rules of the game’ and they embody a ‘feel for the game’ such that they sense the ‘imminent future’ and ‘sense of direction’ of the game (Bourdieu 1990: 82, Fowler 1997: 18). The conditions of the field determine what can be done by actors in the field (Bourdieu 2000, Bourdieu and Wacquant 1992, Grenfell 2008, Webb *et al*. 2002).

Within the field, different actors have different amounts of assets, resources or ‘capital’, and the amount and distribution of such capital determines an actor's place in the field, with actors attempting to profit from their resources. Such capital may be in the form of cultural (e.g. educational credentials, style of speech), economic (e.g. generated wealth), and/or social capital (e.g. resources accrued through social networks) as well as symbolic capital, which takes on this status once it becomes legitimate or recognised by others (Bourdieu and Wacquant 1992).

Bourdieu summarises the interaction between habitus, field and capital in the equation [(habitus)(capital)] + field = practice, essentially stating that practice is shaped by the relationship between an individuals’ habitus and their position in a field within the state of play of that field (Grenfell 2008). Thus behaviours and beliefs of individuals are therefore rational since they follow a ‘practical logic’ arising from these interactions and as a corollary, based on prior experience, resources and an understanding of particular contexts (Bourdieu 1984).

## Methods

This study was a nested qualitative study selecting participants from the Avon Longitudinal Study of Parents and Children (ALSPAC). ALSPAC is a population based cohort study of children born to mothers resident in Avon in the South West of England who had expected dates of delivery between 1st April 1991 and 31st December 1992 (Boyd *et al*. 2013). The aim of this qualitative study was to explore young people's views and experiences around tobacco, alcohol and cannabis use. To this end, the study adopted purposive sampling in order to speak to young people with a variety of substance use behaviours. Between April 2009 and September 2010, participants responded to a questionnaire which included questions relating to their drinking behaviour, including age at first drink and frequency of use. They were also asked whether they had ever smoked a cigarette or used cannabis in their lifetime and in the previous six months. Potential participants were then identified on the basis of responses to these questions. In total, 147 invites were sent out and 28 young people aged between 18 and 20 were recruited (13 male; 15 female). Participants lived in both urban and rural environments, and as far as we are aware, none were friends with each other. Where given, maternal education ranged between none and postgraduate and the majority of mothers were in professional occupations. Participants’ qualifications ranged from A level (n = 17, 61%) to no qualifications (n = 1). There were 9 participants (32%) who had obtained GCSEs (General Certificate of Secondary Education), NVQs (National Vocational Qualification) or national diplomas, although the level of each qualification was not always provided. Half of the participants were in employment or seeking employment and half were in, or were planning to attend, tertiary education.

We note that although the interviews covered the use of all substances, this paper focuses only on views and perspectives around alcohol use, which emerged as a substance used by the majority of participants. Details about the alcohol use of participants is provided in Supplementary Table 1. Informed consent was obtained from participants, and ethical approval for the study was obtained from the ALSPAC Ethics and Law Committee and the Local Research Ethics Committees. The ALSPAC study website contains details of all the data that is available through a fully searchable data dictionary (Avon Longitudinal Study of Parents and Children 2014).

### Data collection

The principle method of data collection was in‐depth interviews (conducted by NJ) which were facilitated by a topic guide, and which explored participants’ views and experiences of alcohol, tobacco and cannabis use. One interview was conducted with a young person and their friend whilst the remainder were conducted on a one‐to‐one basis. Interviews were conducted in participants’ homes, universities or local cafés, and focussed on young people's: initiation into and ongoing use of substances; reasons for use; experiences of drug and alcohol education; understandings of the associated harms, risks and perceptions of risk; outlook on the social context and social networks in which use occurred; and the role of peers, subjective norms, attitudes, beliefs and intentions in shaping substance use. Interviews were digitally‐recorded and transcribed verbatim. Participants provided signed informed consent and received a £10 voucher for taking part. All interview data were fully anonymised, with participant names being changed to preserve anonymity.

### Data analysis

Data analysis was ongoing and iterative, such that early data analyses, and the identification of emergent themes, informed the development of topic guides for subsequent interviews. Transcripts of interviews were imported into NVivo version 10 (QSR International, Brisbane) and analysis was completed using this software. Transcripts were read and re‐read and coded inductively, initially employing open coding to categorise and organise the data. A constant comparative approach was used to identify similarities and differences between accounts, to explore relationships, and to continually refine codes and develop categories and sub‐categories until data saturation was reached with no new data emerging in the final interviews. Notes and memos were written throughout the period of analysis to facilitate this process. Two researchers coded the data independently (GJM, NJ) (owing to staff changes and a need for GJM to become immersed in the data) thus the key categories and themes were corroborated. The major themes identified in relation to the role of peers in young people's alcohol use were: the social context to drinking; peer influence, pressure or selection; safety, trust and responsibility; and judgement and discourse around others. Notably, however, we found that underpinning the study themes was evidence of a deeply entrenched culture around pre‐drinking and drinking. In this way, analysis of the impact of peers could not be considered in isolation and the wider environment and socio‐cultural context needed to be taken into account to more fully understand young people's behaviour. Bourdieu's concepts habitus, field and capital helped to explain the findings by capturing the importance of the cultural context, and by implicitly accounting for the role of previous and ongoing experiences and circumstances throughout adolescence, and the social nature of alcohol consumption. Bourdieu's concepts of the doxa and collusion also capture the importance that young people ascribed to the protection from risk provided by the social group and the need to be responsible or look out for each other – mediated via a mutual understanding and an awareness of the ‘rules of the game’. We note that the links between the data and the theory of Bourdieu were made following data analysis to help explain the findings and to identify possible strategies and/or interventions that could be implemented to reduce the harms associated with adolescent alcohol use.

## Results

### The dialectic of the internalisation of externality and the externalisation of internality

Wider British cultural norms around alcohol were characterised by a tolerance and acceptance from parents around young people's drinking, the ubiquity of alcohol through society, and the integral nature of drinking in the social world. Among young adults in this study, the internalisation of cultural norms and the expected and accepted practice of drinking into the habitus played a critical role in shaping and patterning young people's drinking behaviour and their engagement with the field (here representing the night‐time economy (NTE), in which actors could be considered to be young people, the alcohol industry, and alcohol‐related businesses such as bars and clubs). Owing to this internalisation of a broadly accepting attitude towards drinking, the habitus of young adults became a structure which generated dispositions and inclinations that tended towards an expectation of drinking and a normalisation of the behaviour. Habitus placed drinking as accepted and central to the social world, such that drinking was something which ‘unthinkingly’ occurred. Participants’ reflected an instinctive understanding of the social context and their ‘feel for the game’:

I can't think of a thing that you go out to in the evening except bowling and things like that, where you don't drink, and even bowling you probably do as well, umm yeah, you just kind of do in the evenings, it's what occurs. (ID 1, M, aged 18)

I^1^ Why do you think that most people drink? What do you think is the biggest reason for young people?

R I think it is just a chain reaction. Everyone else is drinking; I'll have a drink as well. I think that's it basically. (ID 14, M, aged 19)

Habitus was attuned to the doxa of the night‐time economy and through the habitus, actions were broadly attuned to each other in line with experiences, precedents and circumstances. As Bourdieu and Wacquant (1992: 127) highlight, ‘when habitus encounters a social world of which it is the product, it is like a fish in water’:

R It's more habit as well because everyone does it and I always do it. You don't even really question why you do it sometimes. It's just something that everyone does.

I It's just normal?

R Yeah it's just normal isn't it? You just on a weekend think oh I will go out tonight, and then you go to a bar because it is such a social area where all your friends go and just drink because you are at a bar. (ID 12, F, aged 19)

With habitus and field interacting in this way, alcohol was clearly pervasive in young people's lives and the habitus of young adults was one where drinking in town centres at weekends was a regular, somewhat patterned activity and a ‘norm’. Drinking was seen as fun rather than problematic or risky in any way. There was little mention of moderate drinking in local pubs, rather, alcohol was widely available and cheap and the culture of drinking revolved around pre‐drinking (‘pre‐loading’ or drinking before going out) and then heavier drinking in bars and clubs in town centres.

I Firstly, if you can tell me about a typical Friday night out, Saturday night out for you? …

R Umm, they kind of vary don't they? We normally either go round a friend's house to get dressed or get changed and so have a couple of drinks at their house, get a taxi into town about eleven, start off in the quieter pubs, work up to three pubs, and then normally get home about 4 o'clock, rather drunk normally which is quite fun. (ID 3, F, aged 18)

I So when you get into town what will you be drinking and where will you go?

R We always start in this one bar because drinks are one fifty, so it's cheap to drink. Usually WKD's and bottles of beer and stuff like that. Then we'll leave there and either go all over, can't really say a specific place because we go everywhere but then I'll drink vodka redbulls all night because they are cheap and just shots of Sambuca and tequila and stuff like that. Anything that's cheap really, in different places the prices vary. (ID 15, F, aged 19)

That was the philosophy. Just go out and get smashed and have a good time. (ID 25, M, aged 19)

However, although drunkenness was a norm for most young adults, the habitus involved strategies for controlling intake and behaviour, reflecting the assimilation and impact of prior histories and experiences and the influence of cost:

I've learnt that if you spend twenty quid on a night out and don't remember much of it it's not actually worth it in the end so I'd rather spend ten and having at least one that you kind of remember and enjoyed it's more worthwhile (ID 9, M, aged 19).

The power of the wider drinking culture in shaping patterns of alcohol use was exacerbated by the family context and a lack of education and awareness around the risks of alcohol consumption. Parents played a primary role in introducing participants to alcohol and subsequently displayed a relatively tolerant approach to their drinking. Furthermore, there was little evidence of information being provided at school:

I Ok if we think a little bit about drug and alcohol education, can you remember if you had any of it?

R Not really. I'm not sure I really had it. (ID 11, F, aged 19)

They [*my parents*] are perfectly happy to buy me booze, they just prefer if I keep it in moderation but they understand university, the whole scene. They understand that things are going to happen. (ID 1, M, aged 18)

Thus, ‘accepting’ school and family contexts contributed to the structuring of habitus, which weighted young people's attitudes and dispositions towards the perceived positive aspects of drinking and the acceptability of the behaviour, contributing to the positive feedback loop with the wider culture of normalised alcohol use.

### The structuring role of habitus

In addition to the wider drinking culture, family influences and social contexts shaping habitus, the internalisation of peer norms was also evident in participants’ accounts, such that peer behaviour influenced individual behaviour. This influence was particularly evident in adolescence during the period of initiation and experimentation with alcohol. At this stage of the lifecourse, participants’ habitus was one of experimentation, excitement, and intoxication, with drinking occurring mostly in the home or outside rather than in dedicated drinking establishments. The desire to start drinking was driven partly by curiosity, but also by a social conformity and a view that ‘everybody else was doing it’:

I What or who had the biggest influence over your drinking then when you started drinking at these sixth form parties? Why did you do it?

R Because my friends were basically.

I Was it pressure to do it?

R No it was my choice; I never got pressured into doing it. I just wanted to fit in with what everyone else was doing. (ID 13, F, aged 19)

Notably, however, the influence of peer behaviour diminished somewhat as young people moved through adolescence. Young people still described an influence of their friends, or a more subtle form of influence characterised by ‘going along with’ the behaviour of their friends, but young people learnt from their experiences, and felt freer to exert their own choices around drinking behaviour:

I What do you think are the main reasons that you drink then?

R I guess it's just because everyone else does, so you tend to just join in don't you. (ID 20, F, aged 19)

Sometimes you go out with the wrong frame of mind I suppose and my friend's bought a pint and then I'll buy a pint, I'll sort of drink it and they say they've finished theirs and I'm like, oh I've got to finish mine. Go and get another pint and I'd try and keep up with them cos they get out more often and I get more drunk than they do. (ID 27, M, aged 19)

I Do they ever try and get you to drink more?

R No. It was always like at the age where we wanted to like, I don't know if it was to fit in or to be cool or whatever. They were just like, ‘oh we've got to do it because they do it’, so you would just do it. But now it's like no one really cares about that. You just drink if you wanna drink and drive if you wanna drive really. (ID 26, F, aged 19)

Many described a greater control over their drinking and an awareness of their individual limits, in order to avoid the negative consequences of intoxication. Thus, young people were making careful choices about the type of drink consumed and pace and volume of drinking, irrespective of their friends’ behaviour. In this way, the habitus changed as young people moved through adolescence to young adulthood to a more measured drinking behaviour, evidencing the way in which habitus indicates that dispositions will differ ‘by social location and trajectory’ or how those with different circumstances, experiences and histories have gained different ways of thinking, feeling and acting (Wacquant 2011). As a result, some peer groups included individuals with a diverse range of drinking patterns. In addition, the choices of those who abstained from drinking alcohol appeared to reflect their distinct abstinent habitus structured by their influences and/or additional responsibilities, rather than an influence of peers and peer norms, *per se*:

I Do you limit what you have? Do you put certain controls in to stop you getting drunk?

R Quite often I work on a Saturday morning as well so I don't go out on Friday nights, I coach rugby, so I have to be up for seven, so it's not a good thing to be coaching when you've got a hangover. Friday is a big no no, Saturday's I am at church in the morning and singing and stuff. (ID 19, M, aged 19)

Thus, the structuring role of habitus was evident since the dispositions produced through the habitus (in part reflecting the embodiment of peer norms) reproduced the structures from which they were derived, reflecting the cycle between habitus and practice.

Among those who attended university, peer behaviour and local norms again influenced the habitus, but to a greater extent, with young people reporting a clear awareness that drinking was ‘the scene’ and integral to university culture. Habitus for these individuals structured more regular and extreme practice reflecting the reported culture of heavy and frequent drinking in these fields and the influence of collective peer behaviour on practice. The importance of social capital was also revealed, since some expressed trepidation and a reluctant engagement in the behaviour in order to accrue social capital and avoid social isolation:

I So when you got there [*to university*], that's when you, did you decide that you were going to start drinking more or was it just something that happened?

R No it's just the culture there really. Everyone goes out all the time and you feel anti‐social if you don't go out and that sort of thing. (ID 13, F, aged 19)

There was also an example of concern expressed around the possibility of losing social capital by failing to act in accordance with the unspoken ‘rules of the game’:

I What about uni, how do you imagine yourself being at uni?

R I'm a bit worried really because I know it's going to be a lot of drinking … I'm worried about what will happen but I think that will make me keep my sensible head on for a bit because I don't want to be labelled badly or anything. (ID 7, F, aged 19)

### Habitus, field and the importance of social capital

The central place of alcohol in social events was clearly evident in participants’ accounts and drinking with friends was equated with fun and enjoyment:

I What do you think are the main reasons that you do drink?

R Umm probably yeah just to, I don't know, I like going out and I like being out with friends … I guess because we all drink, so we are all drinking together, social thing I guess. (ID 11, F, aged 19)

I What is the purpose of getting drunk?

R Umm, I don't know, I guess to have more fun …

I And why do you think that is?

R Being drunk in a group you have more laughs and it's a bit freer so you just have more fun (ID 11, F, aged 19).

The acquisition and maintenance of social capital by young people in the field appeared to be an additional motivator to the practice of drinking, through engagement with existing social networks and/or via the development of new social connections. First, alcohol experimentation and use was viewed as new and exciting and the consequences of excess drinking were experienced together with friends. Young people wanted to join in with the experiences of their friends and to be part of the social group:

When you're in school I think that's quite, well not important but when you're that age it's quite important to do what everyone else is doing, whereas when obviously you get older you realise it's not, I don't know how to explain it … you don't want to feel left out, you don't want all your friends to be having a nice time drinking and laughing around and you just be the only sober one there I think. (ID 2, F, aged 18)

Later in adolescence, participants highlighted how alcohol boosted confidence in social interactions, making them feel less self‐conscious and making the evening out with friends more enjoyable overall:

It is really good socially because you can meet new people and be more confident and just click with people more and it just gives you that boost of confidence and not be self‐conscious and things. (ID 12, F, aged 19)

Greater confidence in socialising played a role in enhancing the likelihood of gaining social capital, providing opportunities to expand the size of the social network and the volume of capital gained.

The social context of alcohol use was such that drinking alone was seen as unusual or cause for concern. Notably, ‘pre‐drinking’, which was frequently an accepted part of a night out, was spoken of as a shared social activity, particularly among girls, providing time to chat among friends and/or to engage in drinking games. Critically, pre‐drinking also enabled alcohol consumption at low cost, thus providing an opportunity to boost social capital in a relaxed environment, whilst simultaneously accounting for economic capital:

I What is the purpose of drinking before you go out?

R I have no idea.

I Is it to get drunk or …

R No not really, it's just to socially have a drink and then we don't tend to buy that many drinks when we go out cos we haven't got that much money.

I So it's cheaper as well?

R Yeah, yeah. (ID 26, F, aged 19)

Taken together, these accounts highlight how the accrual of social capital and the enhancement and subsequent recognition of social status by peers, may be a key driver for young people's drinking practices, enabling them to gain symbolic power and additional prestige.

In contrast to quantitative studies, there was little evidence of individuals selecting friends based on their drinking behaviour, possibly since the shared practice of going out was such that there were opportunities to gain social capital irrespective of individual drinking patterns. Similarly, peer pressure was not involved in decisions to start drinking (see quote above). However, there were isolated instances where young people spoke of peer pressure related to drinking, for instance, in relation to sports culture (e.g. rugby), special occasions, or when drinking more moderately:

If someone said oh no I'm not drinking tonight everyone accepts you are not drinking but when one of them starts drinking, the people that are drinking start putting pressure on them to drinking more and more and more ‘til it gets too much. (ID 17, F, aged 19)

Some also evidently felt an underlying subtle pressure from friends resulting in their use of specific strategies, most frequently driving, to enable them to abstain whilst circumventing any pressure from friends:

I Do you think that you can say no to alcohol?

R Well there's always the get‐out clause with alcohol if I'm driving, especially if you've got a drivers licence you're safe and even if you're not driving and you just don't want to drink you say I'm driving and all of those scare adverts I think have actually got into people's heads that people are allowed to say no to alcohol because they are driving (ID 1, M, aged 18)

Thus, there was evidence that stepping outside of the drinking culture and abstaining without reason might entail perceived risks to social capital, thereby encouraging individuals to use excuses or strategies deemed to be more socially acceptable.

## *Collusio* and accordance with the *doxa*: Safety and protection from risk

Young people's habituses provided the basis for collusion (or ‘an agreement in ways of judging and acting … the basis of a practical mutual understanding’, Bourdieu 2000: 145) via the importance ascribed to friends in providing protection from risk. Friends stayed together and provided a safe unit within which members of the group would look out for each other and make sure that people returned home safely:

I Do you think there are any other kinds of risks involved? How do you manage against any other risks that could potentially be involved with drinking?

R I guess we all look after each other when we are out …

I Is that important to you?

R Yeah I think so. I wouldn't ever go out on my own or with people that I wasn't really, I don't know it's just nice to have people looking out for you and I guess it's all part of the fun as well, we are all there together. (ID 11, F, aged 19)

Participants displayed an accepted way of behaving, evidencing responsibility to each other and protecting each other from risk while attempting to maximise enjoyment and avoid ruining a night out. Some described a shared role of regulating their friends’ drinking to avoid excessive intoxication, while for many, it was accepted that a nominated individual would take responsibility and look after those suffering from injury or sickness. Such practice maintains social capital and accords with tacitly accepted rules of practice within the field. Several participants highlighted the importance of having trust in the peers with whom they drank alcohol, likely owing to a tacit acknowledgement that a friend understood unspoken rules and could be relied upon:

I And is that important, having the trust in a group of friends?

R Mmm. I don't think I would be able to go out with people I don't really know

I Why not?

R Well because every time, like if I go out with a group of friends and then a load of their friends come over that I don't know I'm like ‘ohh I don't trust them, I don't know what they are capable of’ so I normally hold back the drinking, I'll have a couple but I won't get drunk, I will stay the sober one. Because I think ‘well one of us has to be responsible at the moment’ … because I don't know them, they could do something if I get really drunk, like put something in my drinks or something. (ID 3, F, aged 18)

The acknowledgement of the need for a safe unit was particularly evident among women, who were aware of their vulnerability; as were the males in the group who described looking out for their female friends in particular:

R I always walk girls home …

I Why do you walk them home?

R So they are safe, I want to make sure my friends are safe and guy mates I am not too fussed about but there are lots of letchy guys about and there aren't many letchy girls. (ID 8, M, aged 18)

Thus there was ‘mutual understanding’ among individuals within the peer group, and in this way, members of the friendship group acted in line with the *doxa* (defined as the ‘presuppositions of the game’ (Bourdieu 1990)).

## The *doxa*: Judgement and discourse around ‘others’

Young people were clear when friends or others displayed behaviours distinct from their own. First, participants described instances where friends could ruin a night out or make individuals feel vulnerable:

I And when you went out would you be like that with them [*your housemates*]?

R Umm I think I didn't drink as much as them but I did drink enough to feel pretty drunk and I didn't really like it because I didn't feel safe when everyone's running away from you and you don't know how you're going to get home.

I Ok how else did it make you feel?

R Umm I don't know, a bit like gross umm, not very well behaved, for women to act like that I think it's a bit gross. (ID 7, F, aged 19)

However, these were generally seen as unfortunate but acceptable consequences of drinking. Second, many displayed a disapproval and distancing from those who were considered to drink to excess and display distasteful and/or antisocial behaviours:

I just hate seeing, like walking down the street and seeing like girls that are so drunk with like a dress up; like that look to me is like they do that and they think that they're gonna impress boys. And I'm like hmm, if I ever got like that shoot me, I can't bear to be like that. (ID 26, F, aged 19)

Such individuals behaved in a way that neither matched the field of participants nor was aligned with the *doxa*. In line with similar practices and dispositions being produced by the habitus among individuals that occupy close positions in a field (Bourdieu 1984), participants described behaviour within their own peer groups as acceptable, displaying a protectiveness over friends’ practice. Similarly, participants used more extreme benchmarks for drinking within the peer group that were modelled on others who were viewed as more distant:

I How much would you say that you normally drink when you do go out?

R Maybe like three glasses of wine, three ciders and a couple of shots, which isn't very much really, not like most people. (ID 7, F, aged 19)

R There are a couple of friends that are always the drunkest, well not the drunkest but always going to be one of the drunk ones. But they are not drunk, drunk, drunk, drunk, like vomit everywhere. They may be passed out in the cab but they are not, I don't have any friends that are those people. (ID 18, M, aged 18)

## Discussion

In this study, we have applied Bourdieu's concepts of habitus, field and capital to show how the alcohol drinking culture of the UK plays a major role in shaping alcohol use behaviour among young people. Using Bourdieu's equation: ([habitus][capital]) + field = practice, we have described how the internalisation of peer and cultural behavioural norms (‘practice’), alongside historical precedent, accepting family contexts and an absence of information and education, generates a shared habitus among young people that constructs heavy alcohol use as normative and is at home in the night‐time economy (‘field’). The continual interaction between the habitus and this field generates and sustains such practice.

We have also reported how the habitus of young people changes from early adolescence to young adulthood, from one of experimentation, excitement, intoxication and social conformity to one that structures pre‐drinking, drinking and engaging with the NTE as a norm but which involves greater choice and control around intake and behaviour. In addition to the above factors which shape habitus, negative experiences of drinking and the changing nature of peer influence shaped views and practices over the course of adolescence, contributing to the shift in this habitus between adolescence and young adulthood. Our finding supports previous studies, which have highlighted a shift in drinking behaviour during teenage years, from an initial focus on intoxication, to a more experienced and refined drinking culture where young people avoid getting too drunk or losing control, and where drinking customs evolve within friendship groups (Järvinen and Gundelach 2007, Measham and Brain 2005, Percy *et al*. 2011, Szmigin *et al*. 2008).

Looking beyond habitus, we have reported that drinking behaviour was rooted in the social world, with a key motivator to drinking being the possibility of gaining social capital and enhancing status. As Bourdieu describes, individuals, alone or collectively, consciously or unconsciously, invest in developing networks of relationships that can be used in the short or longer‐term; and thus benefit from the assimilated capital of the sum of social networks (Bourdieu 1986). Our findings support those of others, which have similarly highlighted the inextricable link between socialising and alcohol use, and the association between alcohol and lowered inhibitions, social bonding, fun and enjoyment (Coleman and Cater 2005, de Visser *et al*. 2013, Niland *et al*. 2013, Percy *et al*. 2011, Roberts *et al*. 2012, Sheehan and Ridge 2001, Szmigin *et al*. 2008, Townshend 2013) and the role of social and symbolic capital (Järvinen and Gundelach 2007; Lunnay *et al*. 2011). Indeed, one study has suggested that alcohol is seen as a ‘required feature for friendship fun’ with those not drinking feeling alone among friends (Niland *et al*. 2013). Similarly we found that drinking was an unquestioned part of a social event and drinking alone was seen as unusual and cause for concern.

In line with the social nature of drinking, young adults highlighted the importance of feeling trust and safety among friends in the peer group, and acknowledged a shared set of tacitly accepted rules, in Bourdieu's terms ‘an agreement in ways of judging and acting’ underpinning a ‘mutual understanding’ (Bourdieu 2000: 145). Thus they were social actors in the field with a natural understanding of expected behaviour. This resonates with Bourdieu's description of ‘implicit collusion among all the agents who are products of similar conditions and conditionings … each agent finding in the conduct of all his peers the ratification and legitimation (“the done thing”) of his own conduct, which, in return, ratifies and, if need be, rectifies the conduct of others’ (Bourdieu 2000: 145). This collusion was linked to the distancing by some participants to the behaviour of other groups, who failed to act in accordance with the ‘rules of the game’. Disapproval of drunken excess has similarly been observed by others, who report a social stigma associated with losing control as a result of consuming alcohol (Percy *et al*. 2011).

While our findings concur with accounts in qualitative studies, they contrast in some ways with data reported in quantitative studies. The latter have demonstrated that peers play a prominent role in driving alcohol use among adolescents and that the impacts of peers may be mediated by peer selection and/or peer influence. Whilst we found evidence for peer influence, this was in a broader context of the impact of the wider alcohol drinking culture which set alcohol consumption in the centre of adolescents’ social lives. Moreover, we did not find substantial evidence to support peer selection or peer pressure as having a prominent impact on past or current alcohol use, with just a minority of participants mentioning the likelihood of losing contact with friends owing to different patterns of drinking. Friendship groups were mixed and those who drank more moderately were not socially excluded, as has been demonstrated in other studies (Frederiksen *et al*. 2012, Percy *et al*. 2011). This may be linked to the ubiquity of alcohol consumption, such that even in more moderate quantities, the practice of drinking was nevertheless a shared one.

Applying Bourdieu's concepts of habitus, field and capital to our data provides a means of considering possible interventions that may help to prevent short and long‐term harms associated with heavy alcohol use in adolescence and young adulthood. First, as outlined above, Bourdieu highlights how habitus and field continually interact, and how habitus is continually influenced by practice, while habitus simultaneously helps to shape practice and the social world. Bourdieu also uses the term ‘hysteresis’ to describe the result of a disruption in the relationship between habitus and field and a change to the relative value of capital (Hardy 2008). He states: ‘As a result of the hysteresis effect … practices are always liable to incur negative sanctions when the environment with which they are actually confronted is too distant from that in which they are objectively fitted’ (Bourdieu 1977: 78). Applying Bourdieu's thinking thus suggests that a disruption in the field relating to alcohol (i.e. the NTE) would lead to hysteresis and a need for habitus to shift and adapt, hence changing the attitudes and inclinations of young people, and as a corollary, behaviour. This suggests that population‐level policies affecting the NTE would lead to a resultant, albeit gradual, shift in habitus and thus practice. Such a notion is supported by the latest evidence, which highlights that policies that regulate pricing and availability (and thus which affect the NTE) can reduce alcohol‐related harm (Anderson *et al*. 2009, Bosque‐Prous *et al*. 2014, de Vocht *et al*. 2015, Jackson *et al*. 2010). Recent studies also demonstrate that adolescent drunkenness is positively associated with high adult alcohol consumption, risky drinking among adults and community‐level consumption (Bendtsen *et al*. 2013, 2014), suggesting that policies that shift alcohol consumption and norms across the population may also have an impact on adolescent alcohol use.

A major focus of public health policy and practice should therefore be in generating momentum and evidence to contribute to the implementation and evaluation of interventions at the population level. Nevertheless, it is important to note that the lack of information about risk communicated to young people, combined with the importance of family and school‐based experiences in shaping habitus, and the heavy drinking culture of university, also suggests that ongoing adjustment to the habitus of young people will require intervention targeted to the individual, family, school, and university contexts. While young people were aware of the need for trust, safety and responsibility, the longer‐term risks to health associated with alcohol use were not considered relevant and it is likely that these need to be made much more pertinent to young people in order to change behaviour.

## Conclusions

In this study, we have highlighted that adolescent drinking is primarily viewed as a social activity and that friends are integral in drinking experiences. Critically, we found that cultural and peer norms played a prominent role in shaping and normalising alcohol use behaviour. Application of sociological theory to the data suggest that a population‐level approach, comprising alcohol‐control policies that regulate the environment in which alcohol is marketed and consumed should be a primary goal of policymakers and practitioners in order to change behaviour and reduce alcohol‐related harm.

## Supporting information


**Supplementary Table 1.** Alcohol use and drinking pattern of participants.

## Note

1 I: interviewer; R: respondent.
