# Supplementary material for: Among friends: a qualitative exploration of the role of peers in young people's alcohol use using Bourdieu's concepts of habitus, field and capital
Source: Sociol Health Illn. 2016 Aug 30;39(1):30–46. doi: 10.1111/1467-9566.12467 (PMC5244664; doi:10.1111/1467-9566.12467)
Supplement: Supplementary file 1 — Supplementary Table 1. Alcohol use and drinking pattern of participants. [file SHIL-39-30-s001.pdf]

Supplementary Table 1. Alcohol use and drinking pattern of participants

Interview number	Gender	Age	Alcohol use[a]	Drinking pattern[b]
1	M	18	Yes	Non-hazardous
2	F	18	Yes	Hazardous (when goes out but not regular)
3	F	18	Yes	Hazardous
4	M	18	Yes	Non-hazardous (Hazardous earlier in adolescence)
5	M	18	Yes	Hazardous
6	M	18	No (past user)	Non-hazardous
7	F	19	Yes	Hazardous
8	M	18	Yes	Non-hazardous / Hazardous (borderline)
9	M	19	No	Non-hazardous
10	F	19	Yes	Hazardous
11	F	19	Yes	Hazardous
12	F	19	Yes	Non-hazardous (Hazardous periodically and earlier in adolescence)
13	F	19	Yes	Non-hazardous
14	M	19	Yes	Hazardous / Harmful
15	F	19	Yes	Hazardous
16	F	18	Yes	Harmful
17	F	19	No	Non-hazardous
18	M	18	Yes	Hazardous
19	M	19	Yes	Non-hazardous

20	F	19	Yes	Hazardous
21	M	19	No	Non-hazardous (non-drinker)
22	F	19	Yes	Hazardous
23	M	19	Yes	Non-hazardous
24	F	19	Yes	Hazardous
25	M	19	Yes	Hazardous
26	F	19	Yes	Non-hazardous
27	M	19	Yes	Non-hazardous
28	F	20	Yes	Hazardous
**TOTAL CURRENT USERS (n, (%))**			**24 (85.7)**	**Non-hazardous: 13 (46)**<br>**Hazardous: 14 (50)**<br>**Harmful: 1 (4)[c]**

[a] 'No' indicates that the individual does not use the substance and has never experimented with its use.

[b] Grouped into three categories. *Non-hazardous*: No drinking or drinking below safe drinking guideline amounts. *Hazardous*: regularly consuming alcohol over the safe drinking guidelines (3-4 units per day for males and 2-3 units per day for females); including heavy sessional drinking, defined as drinking a large amount of alcohol in a short space of time. *Harmful:* those drinking above recommended limits, and at higher levels than most hazardous drinkers; possibly with evidence of alcohol-related harm. Based on guidance on the consumption of alcohol by children and young people from Sir Liam Donaldson, Chief Medical Officer for England. Department of Health (2009). http://webarchive.nationalarchives.gov.uk/20130107105354/http:/www.dh.gov.uk/prod_consum_dh/groups/dh_digitalassets/documents/digitalasset/dh_110256.pdf (page accessed April 2016). Note that these classifications were based on what was said by participants in the interviews.

[c] Numbers calculated according to lower risk level of alcohol consumption where participants where on the borderline between two categories.
